# Preoperative Cardiac Variables of Diastolic Dysfunction and Clinical Outcomes in Lung Transplant Recipients

**DOI:** 10.1155/2013/391620

**Published:** 2013-09-12

**Authors:** Ajay Yadlapati, Joseph P. Lynch, Rajan Saggar, David Ross, John A. Belperio, Stephen Weigt, Abbas Ardehali, Tristan Grogan, Eric H. Yang, Jamil Aboulhosn

**Affiliations:** ^1^Department of Cardiology, UCLA Medical Center, David Geffen School of Medicine at UCLA, Los Angeles, CA 90095, USA; ^2^Department of Pulmonary & Critical Care, UCLA Medical Center, David Geffen School of Medicine at UCLA, Los Angeles, CA 90095, USA; ^3^Department of Thoracic Surgery and Transplant Surgery, UCLA Medical Center, David Geffen School of Medicine at UCLA, Los Angeles, CA 90095, USA; ^4^Department of Medicine Statistics Core, David Geffen School of Medicine at UCLA, Los Angeles, CA 90095, USA

## Abstract

*Background*. Orthotopic lung transplantation is now widely performed in patients with advanced lung disease. Patients with moderate or severe ventricular systolic dysfunction are typically excluded from lung transplantation; however, there is a paucity of data regarding the prognostic significance of abnormal left ventricular diastolic function and elevated pretransplant pulmonary pressures. *Methods*. We reviewed the characteristics of 111 patients who underwent bilateral and unilateral lung transplants from 200 to 2009 in order to evaluate the prognostic significance of preoperative markers of diastolic function, including invasively measured pulmonary capillary wedge pressure (PCWP) and echocardiographic variables of diastolic dysfunction including mitral *A* > *E* and *A*′ > *E*′. *Results*. Out of 111 patients, 62 were male (56%) and average age was 54.0 ± 10.5 years. Traditional echocardiographic Doppler variables of abnormal diastolic function, including *A*′ > *E*′ and *A* > *E*, did not predict adverse events (*P* = 0.49). Mildly elevated pretransplant PCWP (16–20 mmHg) and moderately/severely elevated PCWP (>20 mmHg) were not associated with adverse clinical events after transplant (*P* = 0.30). Additionally, all clinical endpoints did not show any statistical significance between the two groups. *Conclusions*. Pre-lung transplant invasive and echocardiographic findings of elevated pulmonary pressures and abnormal left ventricular diastolic function are not predictive of adverse posttransplant clinical events.

## 1. Introduction

Nearly three decades have passed since the first successful clinical lung transplant was performed and has become the preferred treatment option for a variety of end-stage pulmonary parenchymal or pulmonary vascular disorders. Due to the shortage of available organs as well as the advancement of disease in most transplant candidates, a full array of preoperative tests is needed in order to consider these patients appropriate applicants. 

As part of the preoperative workup of these patients, investigation of cardiac function with echocardiography and catheterization has been long considered the norm, yet predictors of outcome from these tests are not well defined. The risk posed by cardiac dysfunction must be assessed individually based on severity of disease, presence of end-organ damage, and ease of control with standard therapies [1]. Patients with moderate or severe ventricular systolic dysfunction are typically excluded from lung transplantation; however, there is a paucity of data regarding the prognostic significance of abnormal left ventricular diastolic function or elevated pulmonary pressures.

## 2. Materials and Methods

### 2.1. Study Design

The study was approved by the University of California, Los Angeles (UCLA) Institutional Review Board. All patients who underwent a bilateral or unilateral lung transplant at UCLA Medical Center from 2002 to 2009 were analyzed (394 patients) by chart review in order to evaluate the prognostic significance of preoperative markers of diastolic function, including invasively measured pulmonary capillary wedge pressure (PCWP) and echocardiographic variables of diastolic dysfunction. Diastolic dysfunction was assessed by traditional echocardiographic variables of abnormal diastolic function, including *A*′ > *E*′ and *A* > *E*. Criteria for LV diastolic dysfunction were obtained from the 2009 ASE guidelines [2].

Exclusion criteria included any patients undergoing re-transplant, patients with lack of presurgical echocardiographic or catheterization data performed at UCLA, and patients with systolic left ventricular function less than 40% were excluded. We identified 111 patients who had pretransplant echocardiographic as well as catheterization data performed at UCLA Medical Center. Echocardiographic information was rereviewed by a blinded cardiologist (JA) to ensure the accuracy of the reports. Additionally, pulmonary artery pressures from preoperative catheterizations were analyzed to assess adverse clinical events posttransplant. 

### 2.2. Statistical Analysis

For comparing time until each clinical endpoint between groups, *P* values were computed utilizing Cox Proportional Hazards models. For comparing differences between the nondiastolic dysfunction and diastolic dysfunction groups, *P* values were calculated using the *t*-test for quantitative variables or chi-square test for categorical predictors. If the sample size was too small for the chi-square approximation to be accurate, Fisher's exact test was used instead. The same methods were used for comparing differences among the mean PCWP thresholds. Logistic regression was used to see if clinical endpoints were associated with demographic variables. Error bars were calculated as a 95% confidence interval for proportions. The Kruskal-Wallis test was used for subgroup analysis of primary lung pathology due to the skewed distribution of these variables. If a significant effect was observed, follow-up pairwise Wilcoxon Rank Sum tests with Dunn-Sidak adjusted *P* values were used to test for specific differences between groups. *P* values less than 0.05 were considered statistically significant. All statistical analyses and plots were performed using R (Version 2.13.1) and IBM SPSS (Version 19).

## 3. Results

Subjects ranged from 22 to 70 years of age (62 male and 49 female) and were transplanted for a variety of disease processes including forty-three cases of idiopathic pulmonary fibrosis, twenty-two of chronic obstructive pulmonary disease, twelve of scleroderma, eight of sarcoidosis, eight of usual interstitial pneumonia, eight of isolated pulmonary hypertension, four of cystic fibrosis, four of various rare etiologies, and two of alpha-1 antitrypsin deficiency cases. 

**Figure 1:**
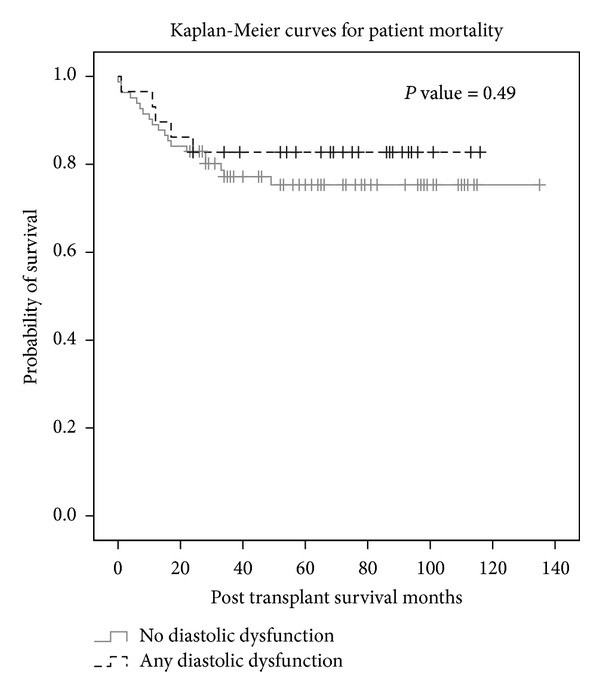
Kaplan-Meier curve shows that there is no significant difference in survival depending on diastolic dysfunction status.

**Figure 2:**
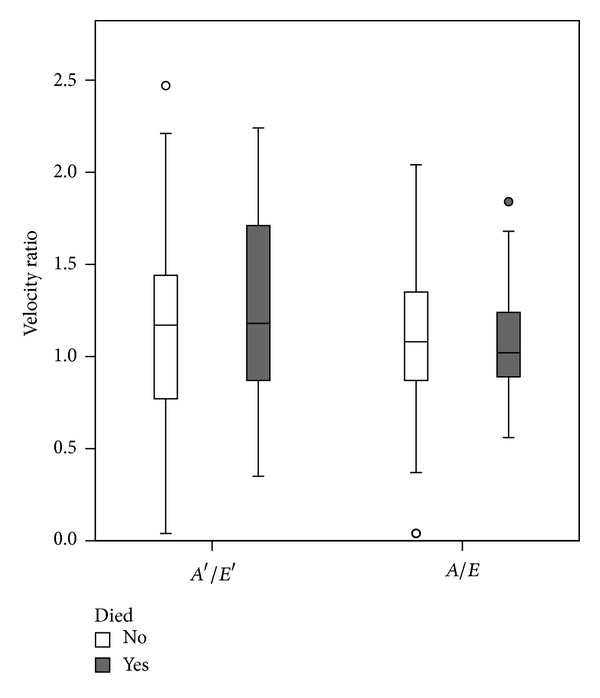
The box plot shows that there is no evidence for a difference in the distribution of the two echocardiographic variables of diastolic dysfunction between survivors and nonsurvivors.

In all, 29 (26.1%) patients met criteria for diastolic dysfunction.  Table 1 presents baseline demographics between both groups. Kaplan-Meier curves were constructed, which did not show statistically significant differences of survival between diastolic dysfunction and normal diastolic function groups for all investigated endpoints (Figure 1). Comparative boxplots were constructed which showed no difference in mortality between each subtype of diastolic dysfunction (Figures 2 and 4). 

**Figure 3:**
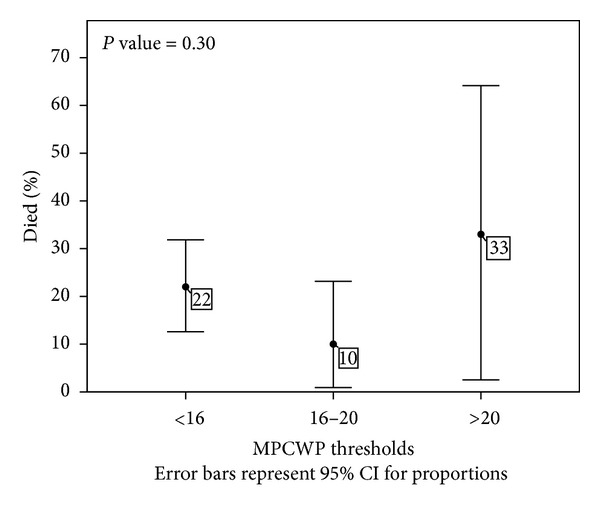
The error bars plot shows that the rate of death does not significantly vary with different mean capillary wedge pressures (MPCWPs) thresholds.

**Figure 4:**
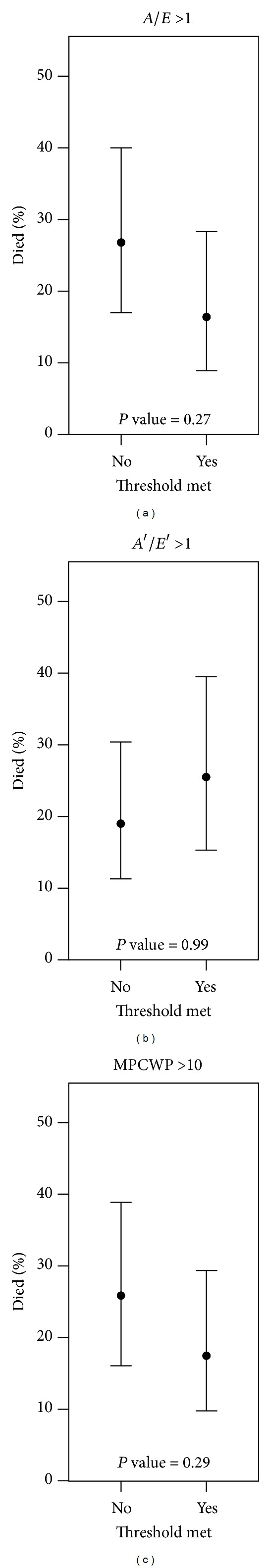
Echocardiographic illustration of abnormal diastolic dysfunction based upon mitral flow and mitral annulus velocity.

**Figure 5:**
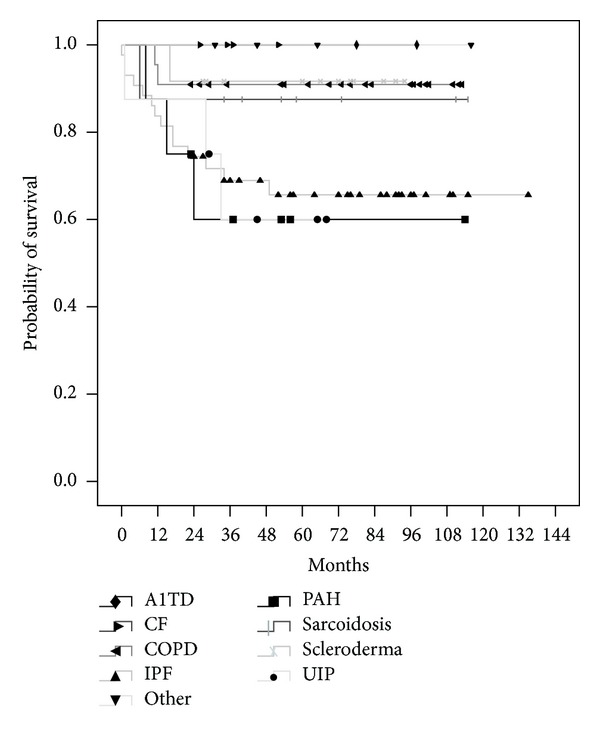
Kaplan-Meier curve shows that there is no significant difference in survival between primary lung pathology groups.

Catheterization data was reviewed and based upon this information, 20 (19.8%) patients were categorized into mildly elevated PCWP (16–20 mmHg) and 9 (8.9%) patients with moderate/severely elevated PCWP (>20 mmHg) (Table 2). The same clinical endpoints as stated above were analyzed in this subset of patients. Mildly and moderately/severely elevated pretransplant PCWPs were not associated with adverse clinical events posttransplant (*P* = 0.30) (Figure 3). Additionally, catheterization profile data between diastolic and nondiastolic dysfunctions did not reveal any statistically significant variables between the two groups, including systolic pulmonary artery pressures (sPAP) (*P* = 0.77), diastolic pulmonary artery pressures (dPAP) (*P* = 0.68), mean pulmonary artery pressures (mPAP) (*P* = 0.84), mean PCWP (*P* = 0.17), cardiac output (*P* = 0.23), cardiac index (*P* = 0.21), and left ventricular ejection fraction (*P* = 0.99) (Table 3). Lastly, a subgroup analysis of primary lung pathology did reveal did not reveal any mortality difference between groups (*P* = 0.176) (Table 4) (Figure 5). Based upon Kruskal-Wallis test with Dunn-Sidak adjusted pairwise comparisons, pulmonary artery pressures were elevated and statistically significant in the pulmonary artery hypertension group in comparison with other groups. Additionally, mean PCWP was statistically lower in the chronic obstruction pulmonary disease group when compared to other groups (*P* = 0.05).

## 4. Discussion

Over the last twenty years, there has been a steady growth in the number of lung transplant operations performed worldwide with concurrent improvement in both short-and long-term outcomes [3]. Various studies have shown some utility in the preoperative workup of certain variables that may exclude certain candidates from lung transplant. Strong negative predictors of one year survival include use of extracorporeal membrane oxygenation, renal failure, age, total bilirubin > 2.0 mg/dL, cardiac index < 2 L/min, steroid dependence, smoking, and body mass index [4–6]. However, pre-existing coronary artery disease has been shown to have acceptable early and medium-term outcomes [7].

Overall, many guidelines have been developed to help risk-stratify candidacy of potential transplant recipients; however, there has been a lack of data regarding short- and long-term outcomes of left ventricular diastolic dysfunction as well as elevated PCWP. Given the scarcity of organs as well as the fact that nearly 20% of lung transplant recipients die within the first year of transplantation, we may be failing to identify those at high risk for severe early complications [8]. 

Some studies have described this dysfunction in nearly 25% to 30% of individuals greater than 45 years of age [9, 10]. On echocardiogram, the mitral inflow velocity profile is used to characterize left ventricular (LV) filling dynamics. The *E* velocity (*E*) represents the early mitral inflow velocity and is influenced by the relative pressures between the left atrium (LA) and LV, which, in turn, are dependent on multiple variables including LA pressure, LV compliance, and the rate of LV relaxation. The *A* velocity (*A*) represents the atrial contractile component of mitral filling and is primarily influenced by LV compliance and LA contractility. In a less compliant heart, a greater proportion of this blood is pushed into the ventricles during atrial systole. In this scenario, the emphasis of ventricular filling during late diastole increases the (*A*) component of the *E*/*A* ratio causing a reversal of the ratio, hence an indication of diastolic dysfunction. Left atrial volume has also been described as an excellent biomarker of the chronicity of diastolic dysfunction and of cardiovascular disease risk [11]. Parameters of diastolic function such as early diastolic velocities measured as *E* prime (*e*′), the *e*′ to late diastolic filling (*A*′) ratio (*e*′/*A*′), and the transmitral to mitral annular early diastolic velocity ratio (*E*/*e*′) [12] have all been shown to predict mortality and cardiovascular events [13, 14]. The early diastolic velocity of the mitral valve annulus (*e*′) reflects the rate of myocardial relaxation. When combined with measurement of the early transmitral flow velocity (*E*), the resultant ratio (*E*/*e*′) correlates well with mean left ventricular end-diastolic pressure [15], hence a marker for LA pressure. 

Left ventricular diastolic dysfunction is a relatively common finding seen on Doppler echocardiography; while multiple studies demonstrate abnormal diastolic function assessment to be associated with increased cardiovascular comorbidity, it can hold varying prognostic significance depending on underlying cardiac ventricular function. It has been shown that mortality is higher in hospitalized patients with depressed left ventricular ejection fractions of less than 39% [16]. Additionally, diastolic dysfunction has been recognized as a strong predictor of mortality in acute myocardial infarction and congestive heart failure [17–20]. However, isolated diastolic dysfunction has significant clinical implications as well. There is much heterogeneity regarding the prognosis of patients with diastolic dysfunction, with mortality ranging from 1.3% to 17.5% [21]. Multiple studies have shown prognostic significance of Doppler indexes of left ventricular diastolic dysfunction where patterns of abnormal relaxation increase the risk of cardiovascular events [22–25]. In one study among 3,107 patients undergoing cardiac catheterization, the small subgroup (1.7%) with diastolic dysfunction, defined as those with high LV end-diastolic pressure and no systolic dysfunction, coronary heart disease or LV dilation had a high risk of future cardiac morbid events [26].

This association between echocardiographic markers of abnormal relaxation and decreased survival, even in those with no history of CHF, suggests that echocardiography may help identify those who are predisposed to adverse outcomes [23]. Given that many lung transplant candidates are excluded with systolic depression, further evaluation of isolated diastolic dysfunction may help identify those at high risk for complications.

There has been much investigation into Doppler echocardiography and comparison with Swan-Ganz catheterization measurements. Studies have shown that hemodynamic data acquired by echocardiography, including estimation of right atrial, pulmonary artery systolic, and PCWPs; cardiac output; and pulmonary vascular resistance, correlate to those of invasive catheterization values [27, 28]. Although correlation is good, estimation of systolic pulmonary artery pressure by echocardiography is frequently inaccurate in patients with advanced lung disease and leads to considerable over-diagnosis of pulmonary hypertension [29, 30]. In addition, technical limitations of the echocardiogram in this patient population often preclude estimating pulmonary artery systolic pressure [30]. Although echocardiography can help estimate preoperative variables for lung transplant candidates, it should be used in conjunction with invasive catheterization rather than replacing it as the sole cardiac assessment modality. Ben-Dor and associates have shown that the prevalence of significant coronary artery disease (CAD) among lung transplant candidates may be low but cannot be accurately predicted by risk factors [31]. The presence of preoperative mild or moderate CAD has been shown not to result in increased perioperative morbidity or mortality or significantly affect the short-term or long-term survival in comparison with recipients without CAD [32]. 

Transplantation remains the only therapeutic option for selected patients with advanced pulmonary arterial hypertension (PAH) who continue to deteriorate despite optimal medical therapy. Given the current shortage of donor organ availability worldwide, there is a need for inclusion of more discriminatory markers of PAH prognosis in donor-lung allocation scores to identify patients at risk and optimize survival to transplantation [33]. Bando et al. [34] and Fang et al. [35] have demonstrated that increased preoperative PAH is an independent risk factor for the development of grade 3 primary graft dysfunction within the first 48 hours after transplant. Additional studies have also shown this correlation between elevated PAH and primary graft dysfunction [36–39]. The relatively high early (30 days and 3 months) mortality in PAH lung transplant recipients in part reflects primary graft dysfunction, a syndrome characterized by noncardiogenic pulmonary edema, hypoxemia, and diffuse alveolar damage within the first 72 hours following lung transplant [37, 40–42]. Because most PAH patients are receiving chronic warfarin, the risk of perioperative bleeding may be increased. Further, following single lung transplant, allograft blood flow is increased in patients with a preoperative diagnosis of PAH compared with emphysema [34, 43]. Hence, estimation of PAH variables is a critical component in the lung transplant workup. Despite these previous studies, our data reveals no end-point difference between patients with normal, mildly elevated, and moderate-severely elevated PAH. Although no statistically significant difference is found, there is indeed a unfavorable trend with regard to mortality in pulmonary hypertension patients. Given the small power in this subgroup analysis, further analysis of this patient population is necessary to access its clinical impact.

No optimal treatment of diastolic dysfunction exists. The objectives of therapy for left ventricular diastolic dysfunction include improvement of preload and afterload hemodynamics [25]. Ace inhibitors and angiotensin inhibitors may provide some additional benefit given their reduction in both pre- and afterload as well as interstitial fibrosis [16]. Additionally, heart rate control is also imperative given its prolongation of left ventricular filling to counterbalance the resistance of inflow due to the stiffened ventricle. 

Our study has several limitations. This is a retrospective single-center review and has inherent limitations associated with all retrospective studies. The lung transplant patients are also highly selected in accordance with our selection protocol. As a result, there may have been a selection bias as the study does not include recipients and experience from other centers. 

In summary, there are many different factors that need to be accounted for when deciding to evaluate and list patients for lung transplantation. A team approach incorporating the surgeon, pulmonologist, and cardiologist is necessary to ensure optimum outcomes in this difficult and challenging group of patients. Pretransplant recipient variables significantly influence early and late survival following lung transplantation, suggesting that some patients face a higher than average risk of mortality during the first year after transplant, as well as severely diminished longer-term survival, that challenges the goals of equitable organs allocation. Further investigation regarding transplant variables are needed to help develop better guidelines, which will ultimately help with optimal utilization of these scarce organs.

The present study demonstrates that prelung transplant invasive and echocardiographic findings of elevated pulmonary pressures, and abnormal left ventricular diastolic function are not predictive of adverse posttransplant clinical events.

## Figures and Tables

**Table 1:** Demographics between diastolic and nondiastolic dysfunction groups. Numbers presented as mean (standard deviation).

	Any diastolic dysfunction (*n* = 29, 26%)	No diastolic dysfunction (*n* = 82, 74%)	*P*-value
Male	66%	52%	0.22
Age at transplant	59.4 (6.2)	52.1 (11)	0.001
Weight (kg)	74.4 (14.2)	73.9 (20.2)	0.91
Diabetes	10%	12%	0.79
Hypertension	28%	35%	0.45
Arrhythmia	24%	27%	0.78
Hyperlipidemia	28%	22%	0.54
MI	0%	0%	—
Ejection fraction	58%	58%	0.98
MPCWP (mmHg)	11.2 (5.2)	14.0 (10)	0.17
*A*/*E*	1.3 (0.3)	0.95 (0.3)	<0.001
*A*′/*E*′	1.6 (0.4)	0.91 (0.4)	<0.001
LAD (mm)	34.7 (5.5)	33.5 (7.2)	0.46
CPBT (min)	207.6 (38.7)	216.8 (60.6)	0.47

MI: myocardial infarction.

MPCWP: mean pulmonary capillary wedge pressure.

LAD: left atrial diameter.

CPBT: cardio-pulmonary bypass time.

**Table 2:** Demographics between mean capillary wedge pressure (MPCWP). Numbers presented as mean (standard deviation).

	MPCWP < 16	MPCWP 16–20	MPCWP > 20	*P* value
Patients	72	20	9	—
Male	53%	55%	67%	0.72
Weight—kg	72.3 (16.0)	75.5 (27.1)	77.0 (17.9)	0.68
Diabetes	13%	10%	11%	0.95
Hypertension	36%	20%	22%	0.32
Arrhythmia	21%	25%	33%	0.67
Hyperlipidemia	28%	5%	33%	0.08
MI	0%	0%	0%	—
CAD	17%	10%	22%	0.66
Ejection Fraction	59%	56%	57%	0.24
Cardiac Output (L/min)	5.2 (1.2)	5.2 (1.3)	4.9 (2.3)	0.82
Cardiac Index (L/min)	3.2 (2.6)	2.8 (0.7)	2.5 (0.8)	0.67
*A*/*E*	1.0 (0.4)	1.1 (0.3)	1.0 (0.4)	0.98
*A*′/*E*′	1.2 (0.6)	1.1 (0.5)	0.9 (0.4)	0.38
LAD	34.0 (6.1)	31.8 (8.9)	37.8 (6.3)	0.10
CPBT (min)	217 (58)	216 (51)	211 (64)	0.95
LAIT (min)	355 (69)	338 (71)	359 (86)	0.65
Died	22%	10%	33%	0.30
Cardiac Death	7%	0%	0%	0.35
PGD 24 hrs	1.7 (1.2)	1.4 (1.0)	1.1 (1.1)	0.45
PGD 48 hrs	1.3 (1.1)	1.3 (1.0)	0.8 (0.7)	0.38
PGD 72 hrs	1.1 (1.0)	1.2 (0.9)	0.9 (0.8)	0.74

MI: myocardial infarction.

CAD: coronary artery disease.

LAD: left atrial diameter.

CPBT: cardio-pulmonary bypass time.

LAIT: lung allograft ischemic time.

PGD: primary graft dysfunction.

**Table 3:** Catheterization and hemodynamic profile of diastolic dysfunction and nondiastolic dysfunction groups.

	Diastolic dysfunction	No diastolic dysfunction	*P* value
Systolic PAP (mmHg)	47.5 (18.9)	47.5 (19.0)	0.77
Diastolic PAP (mmHg)	21.2 (9.9)	22.1 (9.6)	0.68
Mean PAP (mmHg)	30.9 (12.7)	30.3 (13.0)	0.84
Mean PCWP (mmHg)	11.2 (5.2)	14.0 (10.0)	0.17
Cardiac output (L/min)	4.9 (0.9)	5.3 (1.5)	0.23
Cardiac index (L/min/m^2^)	3.6 (4.6)	2.9 (0.7)	0.21
Left ventricular ejection fraction (%)	58%	58%	0.99
*A*/*E*	1.3 (0.3)	0.9 (0.3)	<0.001
*A*′/*E*′	1.6 (0.4)	0.9 (0.4)	<0.001

PAP: pulmonary artery pressure.

**Table 4:** Kruskal-Wallis test with Dunn-Sidak adjusted pairwise comparisons, values presented as mean (standard deviation). Pulmonary artery hypertension (PAH) systolic pulmonary artery pressures (sPAP) are statistically significant.

Variable	A1ATD (*n* = 2)	CF (*n* = 4)	COPD (*n* = 22)	IPF (*n* = 43)	Other (*n* = 4)	PAH (*n* = 8)	Sarcoid (*n* = 8)	Scleroderma (*n* = 12)	UIP (*n* = 8)	KW *P*-value
sPAP (mmHg)	32.0 (4.24)	38.0 (6.16)	37.6 (12.17)	40.6 (14.78)	39.0 (9.20)	73.5 (17.54)	61.4 (20.75)	50.8 (19.13)	61.6 (26.54)	<0.001
dPAP (mmHg)	16.0 (1.41)	18.0 (8.12)	21.7 (7.22)	18.3 (8.98)	21.5 (6.35)	31.3 (7.81)	29.8 (9.87)	20.8 (7.88)	29.5 (13.48)	0.003
mPAP (mmHg)	22.0 (1.41)	24.5 (7.05)	28.1 (7.44)	25.2 (10.52)	27.5 (6.14)	47.1 (10.26)	40.9 (13.62)	32.3 (11.77)	41.9 (19.63)	<0.001
mPCWP (mmHg)	4.5 (6.36)	13.0 (5.29)	14.1 (7.50)	9.7 (12.08)	11.0 (3.56)	13.9 (6.58)	14.4 (4.69)	12.8 (8.90)	15.9 (6.36)	0.05
CI (L/min/m^2^)	3.1 (0.85)	4.1 (0.25)	3.0 (0.47)	3.5 (3.96)	2.7 (0.23)	2.2 (1.18)	3.0 (0.66)	2.6 (0.71)	2.5 (0.44)	0.144
EF (%)	0.54 (0.01)	0.62 (0.03)	0.56 (0.06)	0.58 (0.06)	0.58 (0.06)	0.58 (0.03)	0.60 (0.09)	0.60 (0.02)	0.55 (0.06)	0.068
*A*/*E*	1.07 (0.38)	0.94 (0.43)	1.06 (0.29)	1.09 (0.35)	0.79 (0.57)	0.85 (0.51)	1.03 (0.17)	1.04 (0.52)	1.18 (0.33)	0.729
*A*′/*E*′	1.40 (0.3)	0.47 (0.35)	0.85 (0.72)	1.06 (0.72)	0.72 (0.51)	0.88 (0.49)	0.34 (0.48)	0.99 (0.62)	0.77 (0.59)	0.165
DD (*n*/total)	(0/2) 0.0%	(0/4) 0.0%	(5/22) 22.7%	(10/43) 23.3%	(1/4) 25.0%	(1/8) 12.5%	(1/8) 12.5%	(5/12) 41.7%	(2/8) 25.0%	0.850

sPAP: systolic pulmonary artery pressure.

dPAP: diastolic pulmonary artery pressure.

mPAP: mean pulmonary artery pressure.

mPCWP: mean pulmonary capillary wedge pressure.

CI: cardiac index.

EF: left ventricular ejection fraction.

DD: diastolic dysfunction.
